# Multilayer Reversible Information Hiding with Prediction-Error Expansion and Dynamic Threshold Analysis

**DOI:** 10.3390/s22134872

**Published:** 2022-06-28

**Authors:** I-Hui Pan, Ping-Sheng Huang, Te-Jen Chang, Hsiang-Hsiung Chen

**Affiliations:** 1 Air Command and Staff College, National Defense University, Taoyuan 335, Taiwan; 2 Department of Electronic Engineering, Ming Chuan University, Taoyuan 333, Taiwan; pshuang@mail.mcu.edu.tw; 3 Department of Electrical and Electronic Engineering, Chung-Cheng Institute of Technology, National Defense University, Taoyuan 335, Taiwan; karl591218@gmail.com (T.-J.C.); hhchen1682003@yahoo.com.tw (H.-H.C.)

**Keywords:** high-capacity, multilayer, reversible information hiding, predicted parameters adjustment, dynamic threshold analysis

## Abstract

The rapid development of internet and social media has driven the great requirement for information sharing and intelligent property protection. Therefore, reversible information embedding theory has marked some approaches for information security. Assuming reversibility, the original and embedded data must be completely restored. In this paper, a high-capacity and multilayer reversible information hiding technique for digital images was presented. First, the integer Haar wavelet transform scheme converted the cover image from the spatial into the frequency domain that was used. Furthermore, we applied dynamic threshold analysis, the parameters of the predicted model, the location map, and the multilayer embedding method to improve the quality of the stego image and restore the cover image. In comparison with current algorithms, the proposed algorithm often had better embedding capacity versus image quality performance.

## 1. Introduction

The development of the Internet of Things (IoT) has changed the physical world by overlaying digital information in different areas, including images, video, blockchain, etc. Visual data sources are enhanced greatly to improve visual data management efficiency. However, with communication across the public networks, the safety issue becomes critical [1]. Therefore, reversible information hiding becomes more and more important. It is also referred to as lossless data embedding, which embeds additional information and payload into digital images in a reversible manner [2]. The word “reversible” in this context indicates that one can remove the embedded data to restore the original image. In recent years, numerous principles of the reversible information hiding technology were proposed, such as the difference expansion method [3,4,5,6], histogram method [7,8,9,10,11], difference of the pixel value method [12,13,14], the least significant bit method (LSB) [15,16], dual-image-based schemes [17,18], other reversible information hiding schemes [19,20,21,22,23,24,25], and the prediction-error method [26,27,28,29,30,31,32]. A good information hiding theory provides a significant capacity for embedded secret data that cannot be detected [1,33,34]. For schemes in the transformation domain, the coefficients of the cover and stego images in the space domain are converted into coefficients in the frequency domain through a processing method, such as discrete cosine transform, discrete Fourier transform, or discrete wavelet transform [35,36,37].

The secret data are usually embedded in the LSB of each pixel for the cover image in the classic steganography. This approach is referred to as the LSBs substitution method [15], which slightly modifies initial values without causing obvious noticeable distortion. The difference expansion transform was invented by Tian who proposed the difference expansion (DE) scheme [3] for high embedding capacity. This is a unique reverse information concealment system in terms of high embedding capability and image quality. Alattar extended Tian’s scheme by generalizing the DE approach for all integer transformation [4]. Chang et al. [38] proposed a reversible method that aims to block the truncation coding (BTC) of compressed color images. Each block of a BTC-compressed color image needs three bitmaps and three pairs of quantitation levels for reconstruction. Recently, Chan et al. [39] proposed a method that transformed a coverage image into the frequency domain by using the Haar wavelet digital transformation (HDWT) method. The scheme compressed the high-frequency band coefficients by the Huffman (or arithmetic) coding method and then integrated the compression and secret data into the high-frequency band. Hsu et al. [40] obtained 12 prediction candidates from the neighboring pixels of the integration pixel based on the modelling hypothesis. The final predictive value was selected among those predictive candidates using both the predictive and the original pixel values. Consequently, a more precise final predictive value was achieved.

For large-capacity and multilayer reversible information, if the capacity of the two layers remains insufficient for the quantity of payloads, a third embedding layer is necessary. Therefore, the embedding process continues until there is enough embedding capability for the payload. However, multilayer embedding causes some unanticipated problems. First, the image quality (peak signal-to-noise ratio, PSNR) drops rapidly due to the differences in the embedding layers. Second, the new difference image has a lower integration capability compared to its previous layer [35]. Each embedding layer gradually diminishes the correlation. This condition will happen not only in the coating directions but also in the surrounding area of the pixel. As a result, multilayer embedding schemes may not be effectively exploited for the pixel neighbor’s inherent correlation [26]. Third, the algorithm cannot maintain its performance because the embedding capacity is limited for each layer. In order to achieve a larger embedding capacity to remove these limits, Tian et al. [3] suggested a multilayer embedding scheme. They split the image into pixel pairs and then incorporated some information into each pair. They divided the image into pairs of pixels, then embedded one bit of information into each pair based on the embedding capacity of the DE transform that can reach 0.5 bits per pixel (bpp) for each layer. Unfortunately, the location map itself required 0.5 bpp when it was not compressed. Hu et al. [35] used two embedding directions for the DE-based reversible data hiding. They established a dynamic mechanism that can search and select extensible differences. This mechanism offers equal chances to slight differences between two difference images and effectively avoids the situation of location map. The most differences in the first layer difference image are exhausted. Unfortunately, there is hardly any chance of embedding the slight differences in the second layer difference image. The location map shows if the pairs are developed. The embedding rate is approximately 0.5 bpp in the over- and under-flow location map for each layer. Khanam et al. [21] divided the encrypted image into four parts, and two parts could possess a significant data hiding rate, around 0.11 bpp.

In this paper, we try to deal with the above-mentioned issues. Based on the integer Haar wavelet transform (IHDWT), we calculated the differences in the mean and high-frequency coefficients between the predicted images and the cover images. In addition, we expanded the differences and embedded payload and ancillary information into the expandable differences. In our algorithm, we applied a location map, predicted model parameters, a dynamic threshold, and the multilayer embedding method for improving the stego image quality then restoring the cover image. The experimental results demonstrate the feasibility and validity of our proposed methodology for reversible information hiding. This paper is organized as follows. The prediction-error expansion and dynamic search for expandable difference methods are presented in Section 2. In Section 3, the proposed scheme is depicted in detail. Next, the experimental results are illustrated in Section 4. Finally, we draw conclusions in Section 5.

## 2. Related Works

### 2.1. Prediction-Error Expansion

Thodi et al. [26] proposed the technique for improving distortion performance with low coating capacity and alleviating the problem of capacity control. The authors proposed a reversible data embedding approach, which was called prediction-error expansion. This enhanced technique exploits the inherent correlation of a pixel’s neighborhoods better than the DE scheme [3]. The prediction-error expansion combines the advantages of DE embedding with the better decorrelation abilities of a predictor. This results in a superior data integration capability compared to DE.

**Figure 1:**
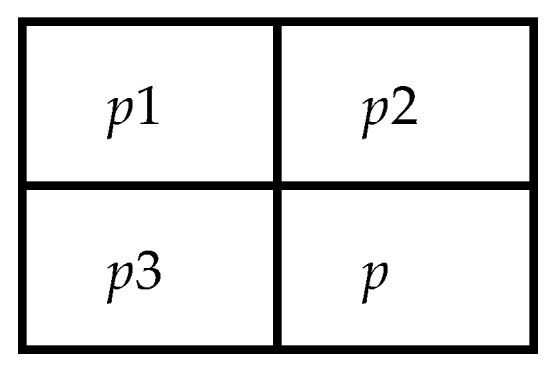
The context of pixel *p*.

This represents a low-complexity algorithm with an inherent edge detection mechanism that is defined as a context of three neighbors. The pixels on the left side, top side, and top-left side of the present pixel are shown in Figure 1.

The output of the predicted value is

(1) $$p^{\prime }=\left\{ \begin{array}{l} \mathrm{if}\;c1\leq \min(p2,\;p3),\;\max(p2,\;p3)\\ \mathrm{if}\;c1\geq \min(p2,\;p3),\;\min(p2,\;p3)\\ \mathrm{otherwise},\;p2+p3-p1 \end{array}\right.$$

where *p* is the present pixel, and *p*1, *p*2, and *p*3 are the context. The predicted intensity, $\hat{p}$, is set to the even integer less than or equal to $\tilde{p}$.

(2) $$\hat{p}=2\times \left\lfloor \tilde{p}/2\right\rfloor$$

Then, the difference between the pixel intensity, *p*, and its predicted intensity, $\hat{p}$, is the prediction error, $p_{e}$, as shown in Equation (3). Embedding a bit, *k*, in $p_{e}$ results in the modified prediction error, ${p_{e}}^{\prime }$, as in Equation (4). The modified prediction errors, ${p_{e}}^{\prime }$, are added to the predicted intensities to create the resulting pixel intensity, and the embedded information is *b*, as shown in Equation (5).

(3) $$p_{e}=p-\hat{p}$$

(4) $${p_{e}}^{\prime }=2\times p_{e}+k$$

(5) $$p\prime ={p_{e}}^{\prime }+\hat{p}=k+p_{e}+b$$

### 2.2. Dynamic Search for Expandable Difference in Horizontal and Vertical Difference Image

**Figure 2:**
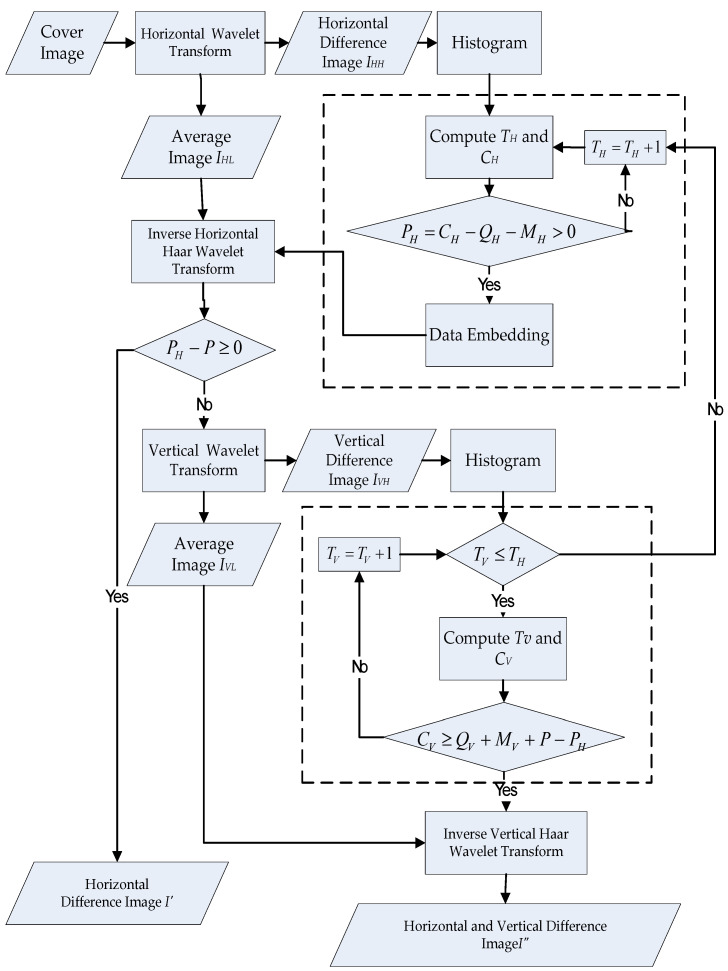
Block diagram of dynamic threshold analysis.

Hu et al. [35] suggested a scheme of extensible locations in the horizontal and vertical difference images. The steps are described as follows and are shown in Figure 2.

1. For a cover image, the integer Haar wavelet transform (IHWT) is just performed in the row direction, and the horizontal difference image, $I_{HH}$, can be obtained. In addition, the histogram of $I_{HH}$ is calculated.
2. Supposing the payload is *P*, and the payload in the row direction is $P_{H}$, the selection of all extendable differences provides spare space for the payload. In addition, there are sufficient locations for the auxiliary data, which consists of the header file, $Q_{H}$, and the location map, $M_{H}$, in the horizontal direction. Assuming the present threshold and the horizontal embedding capacity are $T_{H}$ and $C_{H}$, respectively, then the initial difference selection threshold is zero. If $P_{H}-P>0$, the horizontal capacity under $T_{H}$ is sufficiently large for *P*, and no more locations are required. If $P_{H}-P\leq 0$, the horizontal capacity under $T_{H}$ is not sufficiently large for *P*. Therefore, only the partial payload, i.e., $P_{H}=C_{H}-Q_{H}-M_{H}$, will be embedded in the horizontal direction. Thus, they need the additional embedding capacity and must look into the image of the vertical difference.
3. In Figure 2, the horizontal embedded image, $I^{\prime }$, is obtained by synthesizing $I_{HH}$ and the average image, $I_{HL}$.The integer Haar wavelet transform is performed on $I^{\prime }$ in the column direction to obtain the vertical difference image, $I_{VH}$. In this way, the difference histogram of $I_{VH}$ is calculated.
4. In the vertical direction, supposing the current threshold and the vertical embedding capacity are defined as $T_{V}$ and $C_{V}$, respectively, then $C_{V}=Q_{V}+M_{V}+P-P_{H}$. The authors use another inner search loop to test whether $C_{V}\geq Q_{V}+M_{V}+P-P_{H}$. Once $C_{V}<0$, it means that the vertical difference image can provide enough embedding capability for the remainder of the payload. As a result, one can hide the data and obtain the embedded vertical difference image, ${I^{\prime }}_{VH}$. If the final embedding capacity is larger than zero ($C_{V}>0$), the search round under the current $T_{V}$ fails. We have to increase $T_{V}$ by 1 and repeat Steps (2)–(4) again. Note that, no matter how we increase $T_{V}$, the vertical selection threshold should not be higher than the horizontal threshold, defined as $T_{V}\leq T_{H}.$
5. By converting the Haar wavelet transform, the embedded vertical difference image, ${I^{\prime }}_{VH}$, can be synthesized, as well as the average image, $I_{VL}$, in Figure 2, to obtain the output image, $I^{\prime \prime }$.

## 3. Proposed Scheme

**Figure 3:**
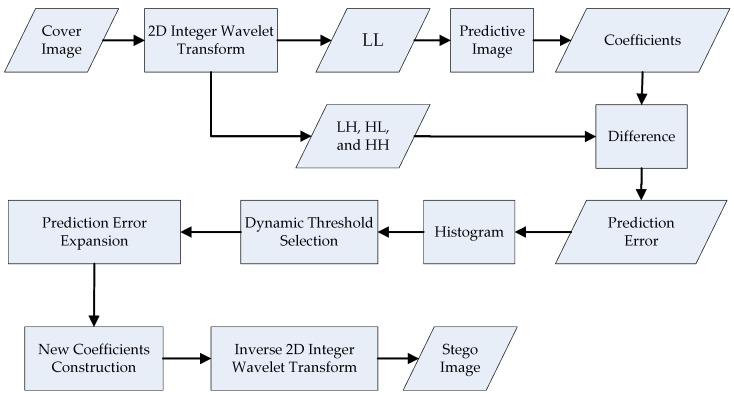
Algorithm of proposed scheme for information embedding.

The algorithm of the proposed scheme for information embedding is shown in Figure 3. Based on the prediction-error expansion and dynamic threshold analysis, we use the integer Haar wavelet transform method to convert the cover image from the spatial domain to the frequency domain. In the first step, we establish a predictive model that uses low-frequency coefficients that retain the most important information.

In the second step, the differences between the medium- and high-frequency coefficients between the predictive image and the original image are calculated. In the third step, we expand the differences and integrate the secret data with evolving expandable differences. In our algorithm, we use the dynamic threshold, predictive parameters of the model, location map, and multilayer embedding method to enhance the quality of the stego image and recover the original cover image. In Section 3.1 and Section 3.2, the proposed schemes for predicted model construction and dynamic threshold analysis are depicted in detail. Subsequently, the adjustment of the predicted parameters for the multiple layers is illustrated in Section 3.3. Finally, details on the proposed information embedding and extraction techniques are described in the Section 3.4.

### 3.1. Predicted Model Construction

The traditional multilayer methods must record the features of each layer for another application or data extraction. In our algorithm, we suggest the identical predicted model for each layer to avoid building large amounts of extra information. However, image quality decreases considerably after the embedding of the first layer because of the use of great differences. Therefore, we present a novel technique for predicted parameter adjustment to construct predictive images for each layer that improve the quality (PSNR) of the stego image.

**Figure 4:**
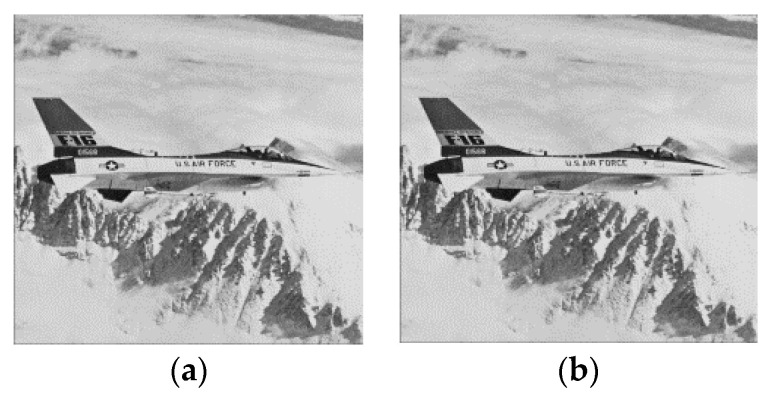
The original and predictive image of “Airplane”; (**a**) original image; (**b**) predictive image.

**Figure 5:**
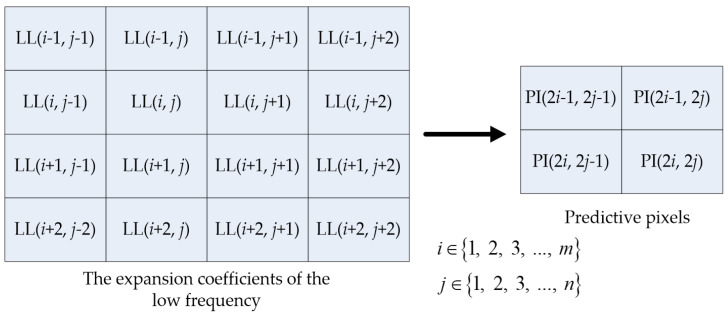
Predicted model construction.

After the integer Haar wavelet transform, low-frequency coefficients contain the most important data from the original image. For this reason, we build the predictive image from low-frequency coefficients (LL), as shown in Figure 4. The steps of the predicted model construction are described as follows:

1. The edge coefficients of the low-frequency coefficients (LL) in the cover image are duplicated.
2. Equations (6)–(9) are used to produce the predictive image PI. In Figure 5, from each low-frequency coefficient, PI (2*i*, 2*j*), PI (2*i*−1, 2*j*), PI (2*i*, 2*j*−1), and PI (2*i*−1, 2*j*−1) denote the four neighboring pixels. For example, the original and predictive images of the “Airplane” image are shown in Figure 4, where PSNR is approximately 30 dB.

(6) $$\begin{array}{l} \mathrm{PI}(2i,\;2j)=\mathrm{LL}(i,\;j),\;\\ \;\;\;\;\;\;\mathrm{for}\;i\in \left\{1,\;2,\;...,\;m\right\},j\in \left\{1,\;2,\;...,\;n\right\} \end{array}$$

(7) $$\begin{array}{l} \mathrm{PI}(2i-1,\;2j)=\\ \frac{\mathrm{LL}(i-1,\;j-1)+\mathrm{LL}(i,\;j-1)+\mathrm{LL}(i-1,\;i+1)}{16}+\\ \frac{\mathrm{LL}(i,\;j+1)}{16}+\frac{3(\mathrm{LL}(i-1,\;j)+\mathrm{LL}(i,\;j))}{8},\;\\ \;\;\;\;\mathrm{for}\;i\in \left\{1,\;2,\;\ldots ,\;m\right\},\;j\in \left\{1,\;2,\;\ldots ,\;n\right\} \end{array}$$

(8) $$\begin{array}{l} \mathrm{PI}(2i,\;2j-1)=\\ \frac{\mathrm{LL}(i-1,\;j-1)+\mathrm{LL}(i-1,\;j)+\mathrm{LL}(i+1,\;i-1)}{16}+\\ \frac{\mathrm{LL}(i+1,\;j)}{16}+\frac{3(\mathrm{LL}(i,\;j-1)+\mathrm{LL}(i,j))}{8},\;\\ \;\;\;\;\mathrm{for}\;i\in \left\{1,\;2,\;\ldots ,\;m\right\},\;j\in \left\{1,\;2\;,\;\ldots ,\;n\right\} \end{array}$$

(9) $$\begin{array}{l} \mathrm{PI}(2i-1,\;2j-1)=\\ \{\begin{array}{c} \begin{array}{l} \frac{1}{4}[\mathrm{LL}(i-1,\;j-1)+\mathrm{LL}(i-1,\;j)+\mathrm{LL}(i,\;j-1)+\\ \mathrm{LL}(i,\;j)],\;............\mathrm{for}\;i\in \left\{1,\;m\right\}\;\mathrm{or}\;j\in \left\{1,\;n\right\} \end{array}\\ \begin{array}{l} \frac{15}{64}(\mathrm{LL}(i-1,\;j-1)+\mathrm{LL}(i-1,\;j)+\mathrm{LL}(i,\;j-1)+\\ \mathrm{LL}(i,\;j))+\frac{1}{128}[\mathrm{LL}(i-2,\;j-1)+\mathrm{LL}(i-2,\;j)+\\ \mathrm{LL}(i+1,\;j-1)+\mathrm{LL}(i+1,\;j)+\mathrm{LL}(i-1,\;j-2)+\\ \mathrm{LL}(i,\;j-2)+\mathrm{LL}(i-1,\;j+1)+\mathrm{LL}(i,\;j+1)],\;\ldots \\ \mathrm{for}\;i\in \left\{2,\;3,\;\ldots ,\;m-1\right\}\;\mathrm{and}\;j\in \left\{2,\;3,\;\ldots ,\;n-1\right\} \end{array} \end{array} \end{array}$$

### 3.2. Dynamic Threshold Analysis

**Figure 6:**
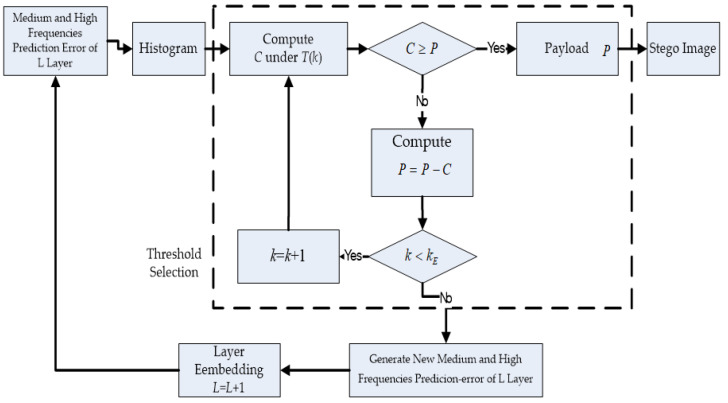
Block diagram of the proposed dynamic threshold analysis.

Numerous studies have been conducted on dynamic threshold analyses [26,30,35,41,42]. The block diagram of dynamic threshold analysis is shown in Figure 6. During data embedding, the steps of the proposed dynamic threshold analysis are described as follows.

1. The multilayer embedding algorithm calculates the prediction error and difference histogram for the coefficients of the medium and high frequencies in the *L* layer. The initial difference selection threshold is zero. We use a difference histogram to describe the statistical distribution of the expandable differences.
2. To avoid unexpected shift, we apply a dynamic threshold analysis. The dynamic threshold, *T*(*k*), is shown in Equation (10). The initial value, *T*(0), is zero, and *k* is the number of the threshold analysis. To achieve a higher image quality, we define the marginal value, *k_E_*. During the experiment, we initially assume eight numbers of the dynamic threshold *T*(1) to *T*(16), as shown in Table 1. Each threshold indicates the value of the prediction error for each layer. We apply the value of initial prediction error, which is zero. Then, we expand both sides starting from the origin. Supposing the payload is *P*, the current selection threshold and the embedding capacity are *T*(*k*) and *C*, respectively. Then, if *C* ≥ *P* and *k* < *k_E_*, the capacity under *T*(*k*) is sufficiently large for *P*. We do not require any more locations. If *C* < *P* and *k* < *k_E_*, this implies that the difference image cannot provide sufficient embedding capacity for the rest of the payload ($P^{\prime }=P-C$). We increase the parameter of threshold, *k,* by 1 and repeat the previous steps. If we find *C* < *P* and *k* < *k_E_*, we must calculate the prediction error and histogram of the medium- and high-frequency coefficients in the next layer.
  (10) $$T(k)={\left(-1\right)}^{k-1}\times (k-1)+T(k-1),\;k\in \left\{1,\;2,\;\ldots ,\;k_{E}\right\}$$
3. If the payload size continues to increase, this method cannot satisfy the capacity requirement in the single-layer embedding, whereas the multilayer embedding method is able to do so.

### 3.3. Predicted Parameter Adjustment of Multiple Layers

To obtain a higher image quality, we not only perform dynamic threshold analysis but also define the predicted parameter adjustments of the multiple layers, as shown in Equation (11). Hence, we adjust the amount of difference image pixels as little as possible to maintain the embedding distortion to the lowest degree. We use the one-level integer Haar wavelet transform to discover the medium-frequency coefficients and high-frequency coefficients, PIH(i), of the predictive image. We define *i* as the number of the predicted parameter, *a* is the scaling, $T_{E}$ is the value of the marginal threshold, and *b* is the shift of the original predicted parameter. The parameter new_PIH(i) is the novel predicted parameter after performing the adjustment.

(11) $$\mathrm{new}\_\mathrm{PIH}(i)=\left\{ \begin{array}{l} \left\lfloor a\times \mathrm{PIH}(i)\right\rfloor +\left\lfloor b\times T_{E}\right\rfloor ,\;\mathrm{for}\;\mathrm{PIH}(i)\geq 0\\ \left\lfloor a\times \mathrm{PIH}(i)\right\rfloor -\left\lfloor b\times T_{E}\right\rfloor ,\;\mathrm{for}\;\mathrm{PIH}(i)<0 \end{array}\right.$$

For example, in the “Lena” image, we assume the marginal threshold, $T_{E}$, is four. We use two layers for data embedding. As we select different values of *a* and *b*, different PSNRs are obtained. If we select smaller values of *a* and *b*, larger PSNRs are obtained; however, fewer secret data are embedded. Considering more data for the embedding, the range of *a* is from –1 to +1, and the range of *b* is from –0.5 to +0.5; however, the value of PSNR is not optimal. While *a* ≤ –0.5 and *b* ≤ –3, a higher PSNR can be generated; however, the embedding rate falls gradually. To obtain a better embedding rate or image quality, we adjust the predicted parameters to achieve our goal.

### 3.4. Secret Data Embedding and Extraction

The embedding process involves calculating the prediction error (PE) from the vicinity of a pixel and embedding the information with the expanded prediction error. We utilize the one-level integer Haar wavelet transform to discover the medium-frequency coefficients and high-frequency coefficients, *x,* of the original image. After the construction of the predictive image, we perform the one-level integer Haar wavelet transform from the spatial domain into the frequency domain and find the medium-and high-frequency coefficients, $\hat{x}$ (HL, LH, and HH). We apply Equations (3)–(5) and find the prediction error ($p_{e}=x-\hat{x}$) and the resulting pixel intensity ($x^{\prime }={p_{e}}^{\prime }+\hat{x}$). During the secret data extraction, we compute the difference between the intensity of the pixels, $x^{\prime }$, and the predicted intensity, $\hat{x}$, of the stego image to obtain the modified ${p_{e}}^{\prime }$. Based on Equation (12), we find the number of values of the prediction error, $p_{e}$, and embedded information, *b*. Finally, we complete the procedures of reversible data hiding.

(12) $$p_{e}=\left\lfloor \frac{{p_{e}}^{\prime }}{2}\right\rfloor ,\;b={p_{e}}^{\prime }-2\times \left\lfloor \frac{{p_{e}}^{\prime }}{2}\right\rfloor$$

#### 3.4.1. Secret Data Embedding

For blind data extraction and the loss-free retrieval of the original image, we use the header file to save all embedding parameters for the reversibility of the calibration process. While hiding secret data, we should build the header file and the over- and under-flow location map. The total bits of header file are shown in Table 2. The header file includes the number of layers, *L*, the predicted parameters, PIH(i), the marginal value, *k_E_*, information on applying the location map, the length of the location map, and the number of embedded payloads in the last layer. If the localization map application information reads “0”, it shows that the header file will not include the length of the location map and the over- or under-flow location map. However, at the decoder, we are unable to extract the hidden data if we assume such an embedding scheme. Actually, without the location map and embedding settings, we cannot know where the selected differences are. This means that even if we have determined the binary flow along with the extensible locations to hide the secret data we need to make this kind of hiding process easy for extracting data blindly.

**Figure 7:**
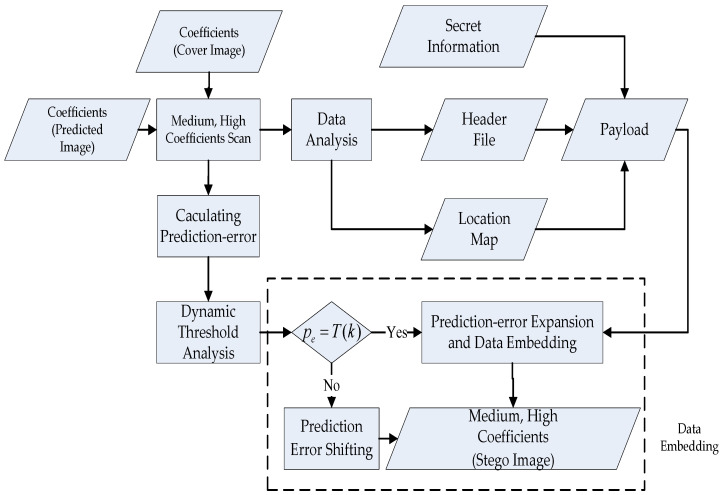
Block diagram of secret data embedding.

The block diagram of data embedding is shown in Figure 7. During secret data embedding, we propose the dynamic threshold analysis. We utilize the one-level integer Haar wavelet transform to find the medium- and high-frequency coefficients, *x* of the cover image and $\hat{x}$ of the predicted image. If the prediction error, $p_{e}=x-\hat{x}$, and the dynamic threshold *T*(*k*) are equal, we perform data embedding, which results in the new value, $x^{\prime }=x+p_{e}+p$, of the medium- and high-frequency coefficients. If the prediction error, $p_{e}$, and the dynamic threshold, *T*(*k*), are not equal, we perform PE shifting, as shown in Equation (13).

(13) $$x^{\prime }=\left\{ \begin{array}{l} x+T(2\times \left\lfloor (k-1)/2\right\rfloor +1)+1,\;\ldots \\ .....\mathrm{if}\;p_{e}>T(2\times \left\lfloor (k-1)/2\right\rfloor +1)\\ x+T(2\times \left\lfloor k/2\right\rfloor ),\;...................\\ .....\mathrm{if}\;p_{e}<T(2\times \left\lfloor (k)/2\right\rfloor ) \end{array}\right.$$

#### 3.4.2. Information Extraction

**Figure 8:**
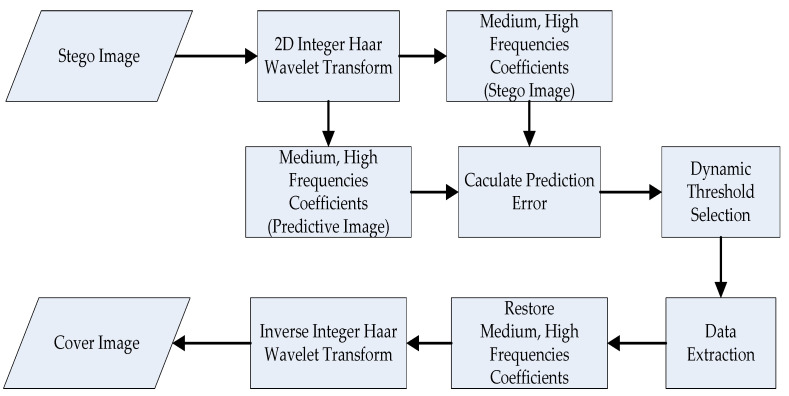
Block diagram of information extraction.

The flow chart for extracting information is presented in Figure 8. During information extraction, we also perform the dynamic threshold analysis and use the one-level integer Haar wavelet transform to find the medium-frequency coefficients and high-frequency coefficients, $x^{\prime }$, of the stego image. After decomposing the integer Haar wavelet transform, we construct the predictive image from the low-frequency (LL) coefficients. At this time, the same dynamic threshold is used for extracting the header file to decide how to extract the secret data and their length. Then, we employ the inverse integer Haar wavelet transform to restore the cover image. The pixels of an image must be in the range of gray levels to avoid the scenarios of under- and overflow. The algorithms for restoring the cover image are described in Equations (14)–(17).

(14) $$\begin{array}{l} 0\leq f(2i,\;2j)=\mathrm{LL}(i,\;j)-\left\lfloor \frac{\mathrm{LH}(i,\;j)}{2}\right\rfloor -\\ \left\lfloor \frac{\mathrm{HL}(i,\;j)-\left\lfloor \mathrm{HH}(i,\;j)/2\right\rfloor }{2}\right\rfloor \leq 255 \end{array}$$

(15) $$\begin{array}{l} 0\leq f(2i,\;2j-1)=f(2i,\;2j)+\\ \mathrm{HL}(i,\;j)-\left\lfloor \frac{\mathrm{HH}(i,\;j)}{2}\right\rfloor \leq 255 \end{array}$$

(16) $$\begin{array}{l} 0\leq f(2i-1,\;2j)=\mathrm{LL}(i,\;j)+\mathrm{LH}(i,\;j)-\left\lfloor \frac{\mathrm{LH}(i,\;j)}{2}\right\rfloor \\ -\left\lfloor \frac{\mathrm{HL}(i,\;j)+\mathrm{HH}(i,\;j)-\left\lfloor \mathrm{HH}(i,\;j)/2\right\rfloor }{2}\right\rfloor \leq 255 \end{array}$$

(17) $$\begin{array}{l} 0\leq f(2i-1,\;2j-1)=f(2i-1,\;2j)+\mathrm{HL}(i,\;j)+\\ \mathrm{HH}(i,\;j)-\left\lfloor \frac{\mathrm{HH}(i,\;j)}{2}\right\rfloor \leq 255 \end{array}$$

## 4. Experiment and Discussion

**Figure 9:**
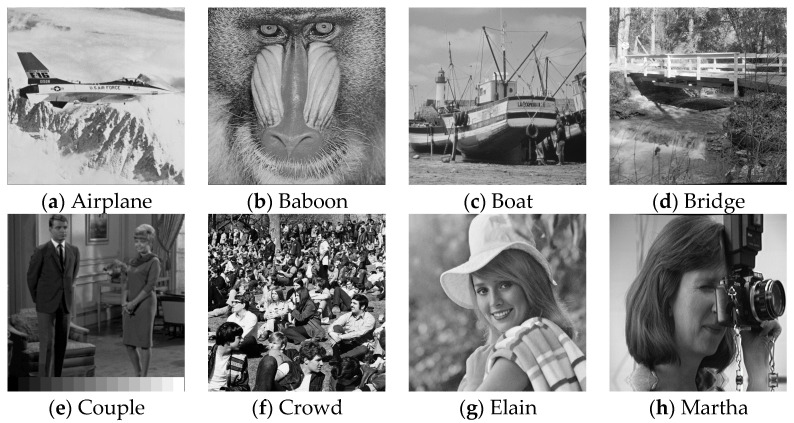

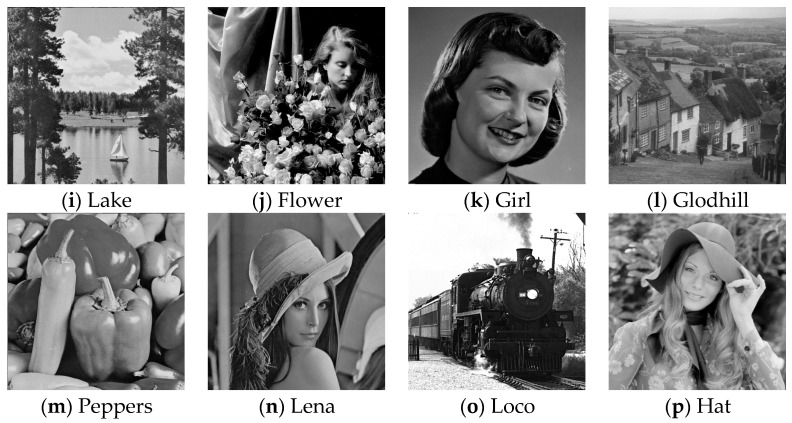
Cover images are shown in (**a**–**p**).

We implemented the proposed reversible information hiding scheme using MATLAB software. In the experiments, we used 16 grayscale images with the size of 512 × 512 × 8 bits. To demonstrate the efficacy of the proposed scheme, we tested it on a variety of images. The images included “Airplane”, “Baboon”, “Boat”, “Bridge”, “Couple”, “Crowd”, “Elain”, “Martha”, “Lake”, “Flower”, “Girl”, “Goldhill”, “Peppers”, “Lena”, “Loco” and “Hat” images, which are are shown in Figure 9a–p, respectively. These images were obtained from open-source image databases (Image Processing Place, https://www.imageprocessingplace.com/root_files_V3/image_databases.htm, accessed on 18 March 2022). They indicated various textural complexities. The stego image quality was measured by PSNR.

In extracting the information, the existence of secret data may be determined by comparing the differences between the extracted data and the original data. The measure of the bit error rate (BER) is indicated by

(18) $$\mathrm{BER}=\frac{\sum_{i=1}^{m}d_{i}⊕d_{i}{}^{\prime }}{m}$$

where $d_{i}$ and ${d^{\prime }}_{i}$ are the original information and the extracted information, respectively. The *m* means the data size, and ⊕ is the XOR operation between the two datasets. For the imperceptible capability, a quantitative index, the mean square error (MSE), and PSNR, are employed to evaluate the differences between a cover image, *p*, and a stego image, $p^{\prime }$, and *N* is the total image size. The MSE and PSNR are defined by

(19) $$\mathrm{MSE}=\frac{1}{N}\sum_{i=1}^{N}{\left(p\prime_{i}-p_{i}\right)}^{2}$$

(20) $$\mathrm{PSNR}=10\times \log_{10}\left(\frac{255^{2}}{\mathrm{MSE}}\right)$$

**Figure 10:**
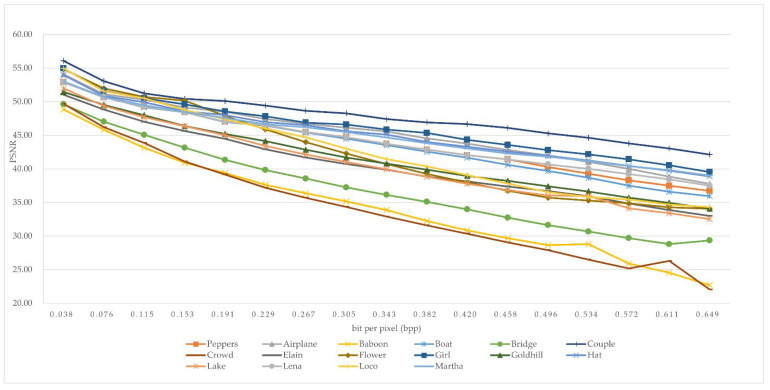
The PSNRs with different bpps.

We made 200,000 bits of secret data and stored them in each grayscale image. The secret data were generated by the MATLAB function round(rand(1,x)), where *x* indicates the length of the secret dataset. The PSNRs with the different bpps of the 16 grayscale images are shown in Table 3. According to Table 1, there are 16 threshold numbers that can be extended and decided for use by *T =* 0, 1, −1, 2, −2, 3, −3, 4, −4, 5, −5, 6, −6, 7, −7, −8. Figure 10 shows the graph of PSNRs with 17 different bpps from 0.038 bpp to 0.649 bpp. The vertical axis indicates the PSNR values, and the horizontal axes shows the bpp values. Compared to the experimental data, the highest PSNR was “Couple” from 56.08 to 42.14. On the contrary, the lowest PSNR was “Baboon” from 56.08 to 22.69. 

Almost all of them can be hidden with only two layers. Because “Baboon” and “Crowd” images have more high-frequency signals, even if four layers are used, the complete message cannot be hidden. In addition, the “Bridge” and “Lake” images used three layers to hide the secret message. The payloads (embedded data and additional information) with different layers and of 16 grayscale images are shown in Table 4.

It was difficult to distinguish between the stego images with human ees. The experiment demonstrates the better transparency of the proposed system. The extracted secret data from the 16 stego images with BER were zero. This means that the reversible data algorithm is a perfect embed and extraction process.

The information of data embedding is shown in Table 5. The parameter *A* is the half value of the variable *a* in Equation (12). It represents the scale of the original value of the predicted image, while *B* is the integer of the value $\left\lfloor b\times T_{E}\right\rfloor$, which represents the shifting of the original predicted parameter. The parameter *k* is the number of the threshold analysis. The phenomena of over- or under-flow were not generated after the data embedding, and the header file did not include the length of the location map on the “Lena” and “Airplane” images. On the “Boat” image, we only used 16 bits of the location map (8 bits of row direction and 8 bits of column direction) to present the underflow of one pixel. The experimental results demonstrate that the data can be extracted without any original image. It can be found that after the parameter revision between different levels, the PSNRs of different information embedding amounts oscillate. As a result, the better PSNR can be obtained under the selection of the better layers and parameters.

**Figure 11:**
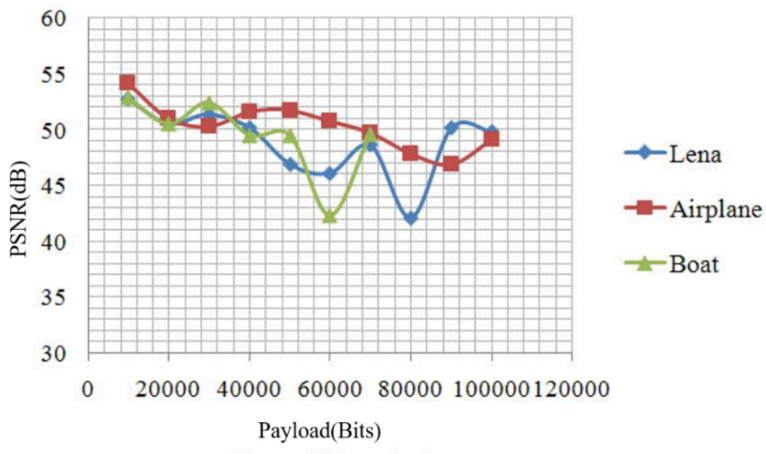
Payload and corresponding PSNR of the “Lena”, “Airplane”, and “Boat” images.

The payload and corresponding PSNR of the “Lena”, “Airplane”, and “Boat” images are shown in Figure 11. We performed 10,000 to 100,000 bits of data embedding. During the secret data embedding, if we wanted to embed the secret data with 60,000 bits, the PSNR of the “Airplane” and “Boat” images were 52.24 and 43.52 dB, respectively. Because the “Airplane” image had more texture complexity than the “Boat” image, the PSNR of the “Airplane” image was significantly better than the “Boat” image. Therefore, how to decrease prediction error and avoid under- and over-flow scenarios will be crucial in the future.

Chang et al. [43] suggested a reversible image approach based on the high payload rate. This performance could be obtained by transforming a spatial domain coverage image into a frequency domain using the HDWT method. It was followed by an adaptive arithmetical coding method for coding HDWT coefficients in a high-frequency band. The quantitative outcomes measured are presented in Table 6. As can see in Table 6, the payload (embedded data and additional information) and PSNR by the proposed method have shown significantly better performance compared to Chang’s method.

We directly used the data provided by Hung et al.’s [27], Hong et al.’s [44], Luo et al.’s [45], and Cai et al.’s [28] methods. Due to space limitation, only parts of the comparison results against their methods are shown. In Ref. [27], the authors offered reversible data hiding for the predictive-error expansion ability based on a multilayer perceptron. It was an artificial neuronal network widely used in numerous applications. They used the correlation between pixel values in the image and adjacent pixels to obtain a multilayer perceptron that could obtain more accurate pixel prediction results. In Ref. [44], Hong et al. proposed the reversible data hiding method using prediction and histogram shifting. A base set of pixels was used to improve predictive accuracy, which could increase the payload. In order to enhance image quality, a threshold was used to select only the low variance blocks to be integrated into the process. In Ref. [45], Luo et al. proposed a reversible watermarking scheme using an interpolation technique that could integrate a large amount of secret data in images with imperceptible editing. They applied the interpolation error and the difference between the interpolation value and the corresponding pixel value to integrate bit “1” or “0” by additively extending it or leaving it unaltered. In Ref. [28], a 3D error-prediction histogram was generated by calculating each overlapping error-prediction triple. Afterwards, the data embedding was counted with a specially designed reversible cartography.

By modifying the pixels slightly, a high image quality was maintained. Consequently, Table 7 shows that although the payload reached 100,040 bits, the corresponding PSNR of the “Lena” image did not drop significantly (49.85 dB). Luo’s method did not use the “Boat” image for testing. In our proposed scheme, the payload was up to 70,040 bits, and the corresponding PSNR of the “Boat” image was 49.62 dB. In the “Airplane” image, the PSNR was 49.70 dB while the payload was 70,042 bits, and the PSNR was 49.14 dB while the payload was 100,042 bits. The embedding capacity and image quality obtained by the proposed method have demonstrated better performance than those obtained by the above methods. Finally, the obtained results are described as follows.

- Increasing the security of data embedding:
  We proposed the predicted model construction using integer Haar wavelet transform decomposition. Then, we constructed the predicted image from low-frequency coefficients (LL). If someone attempts to steal the embedding information, they must use the key of the predicted model and parameters to extract the perfect information. Otherwise, some imperfect information or random unrecognizable characters and numbers may be found.
- Improving the payload of the location map:
  After the integer Haar wavelet transform decomposition, the low-frequency image is one quarter compared to the original image. For a 512 × 512 image, the location map size is 256 × 256. Hence, the embedding rate is 0.25 bits per pixel (bpp) when it is not compressed compared with Tian’s method [3] where the embedding rate is 0.5 bpp. Evidently, not all pairs can be used for data hiding. The location map indicates whether the pairs are expanded. Compared with Hu’s method [35], where the embedding rate is near 0.5 bpp with embedding the over- and under-flow location map for one layer, our proposed scheme can reach to 0.75 bpp, at best, in one layer.
- To improve the quality (PSNR) of the stego image. There are some lists for our proposed method:
  First, the dynamic threshold analysis calculates the PE and histogram of the median and high-frequency coefficients in each layer. The initial difference selection threshold is zero. When the payload is completely embedded into the cover image, the dynamic threshold analysis and other unexpected shifts are stopped. Therefore, the quality of the stego image can be improved. Second, we constrict the predicted model to low-frequency coefficients (LL). The low-frequency coefficients contain the most important information of the original image. During the secret data embedding and extraction, we not only never change the predictive image but also perfectly reconstruct it. After constructing the predicted image, we only find the median and high-frequency coefficients to finish the reversible data hiding and increase the quality of the stego image. Third, we improve the location map size such that we not only can use significantly lower extra information during the same data embedding but can also embed more payload. As we can see in Table 6, Chang et al. [43] used a more significant amount of extra information for performing reversible data hiding. Fourth, to obtain a higher image quality, we not only perform the dynamic threshold analysis but also define the predicted parameters of the multiple layers.
- After the integer Haar wavelet transform decomposition, we duplicate the edge coefficients of the low-frequency coefficients (LL) of the cover image to construct the predicted image. The detected features of the different texture complexities of the predicted image are important. For example, during the data embedding, we set the threshold to 8 and the predicted parameters to *a* = 1 and *b* = 0 with one layer embedding. Under the same conditions (the embedding rate is 0.38 bpp), the PSNRs of the “Baboon” and “Airplane” image were 32.24 and 44.52 dB, respectively. The PSNR of the “Airplane” image was significantly better than that of the “Baboon” image. Thus, a method that maintains the high-frequency coefficients, decreases prediction errors, and avoids under- and over-flow scenarios is crucial for future endeavors.
- If we use different predicted parameters, sometimes we can obtain better PSNRs but worse embedding rates, and at times we may find better embedding rates along with worse PSNRs. In Figure 11, under the same conditions, the embedding rate was 0.4 bpp, and we used the fourth and sixth parameters to determine that the PSNRs were 48.378 and 40.393 dB, respectively. An algorithm capable of searching the best parameters will be a key research endeavor in the future. In addition, a quick decision method for the selection of predicted parameters may be the next step for our research. Finally, this approach can be applied to copyright protection and digital archives.

## 5. Conclusions

We present an improved high-capacity and multilayer reversible information hiding technique. The proposed scheme uses the predicted model to improve the payload of the location map. Because we improved the location map size, we can use a significantly lower amount of extra information during the same data embedding. However, we can also embed an increasing amount of secret data. Subsequently, in the proposed algorithm, we constructed the dynamic threshold analysis, the predicted parameters, the location map, and the multilayer embedding method for enhancing stego image quality and restoring the cover image. Depending on the experimental results, the proposed reversible scheme offers greater capacity and better image quality for stego images.

## Figures and Tables

**Table 1:** Dynamic threshold *T*(1) to *T*(8).

*T*(1)	*T*(2)	*T*(3)	*T*(4)	*T*(5)	*T*(6)	*T*(7)	*T*(8)
0	−1	1	−2	2	−3	3	−4

**Table 2:** Total bits of header file.

	*L*		Predicted Parameters	*k_E_*	Apply Location Map	The Length of Location Map	The Number of Embedded Payloads in the Last Layer	Total Bits
Does the last layer apply location map?	Yes	4	16	12	1	16	16	65
	No	4	16	12	1	0	16	49
Other Layer	----		16	12	----	----	----	28

**Table 3:** The PSNRs with different bpps.

**bpp**	**Peppers**	**Airplane**	**Baboon**	**Boat**	**Bridge**	**Couple**	**Crowd**	**Elain**
0.038	52.89	54.09	48.85	52.99	49.62	56.08	49.69	51.06
0.076	50.64	51.11	45.84	50.66	47.02	53.05	46.19	48.83
0.115	49.27	50.28	43.14	49.18	45.06	51.24	43.84	47.01
0.153	48.43	49.15	40.91	48.43	43.16	50.41	41.09	45.62
0.191	47.04	48.50	39.38	46.99	41.35	50.09	39.16	44.49
0.229	46.50	47.41	37.63	46.45	39.83	49.41	37.20	42.91
0.267	45.48	46.73	36.37	45.40	38.55	48.64	35.69	41.69
0.305	44.74	46.12	35.17	44.50	37.24	48.27	34.35	40.72
0.343	43.74	45.57	33.88	43.53	36.13	47.40	32.90	39.87
0.382	42.87	44.53	32.24	42.56	35.11	46.93	31.59	38.86
0.420	42.03	43.75	30.84	41.67	33.97	46.67	30.34	38.14
0.458	41.42	42.82	29.70	40.61	32.75	46.10	29.05	37.39
0.496	40.29	42.01	28.65	39.69	31.63	45.28	27.90	36.69
0.534	39.28	41.02	28.80	38.68	30.67	44.63	26.50	35.93
0.572	38.29	40.00	25.90	37.49	29.69	43.78	25.18	34.85
0.611	37.50	38.85	24.55	36.59	28.80	43.04	26.31	33.86
0.649	36.73	37.77	22.69	35.94	29.35	42.14	22.05	33.00
**bpp**	**Flower**	**Girl**	**Goldhill**	**Hat**	**Lake**	**Lena**	**Loco**	**Martha**
0.038	54.83	54.97	51.44	53.97	51.98	52.89	54.95	53.00
0.076	51.98	51.62	49.53	50.87	49.35	50.64	51.55	50.71
0.115	50.70	50.53	47.99	49.91	47.69	49.27	50.51	49.48
0.153	50.16	49.60	46.37	48.57	46.35	48.43	48.76	48.47
0.191	47.90	48.54	45.21	48.02	45.02	47.04	47.43	47.56
0.229	45.87	47.81	44.12	46.93	43.41	46.50	46.00	46.64
0.267	43.98	46.90	42.84	46.52	42.13	45.48	44.69	46.19
0.305	42.29	46.59	41.69	45.61	41.03	44.74	43.00	45.46
0.343	40.72	45.84	40.79	45.09	39.96	43.74	41.44	44.65
0.382	39.26	45.34	39.89	44.02	38.81	42.87	40.38	43.83
0.420	37.97	44.32	38.95	43.29	37.80	42.03	39.06	43.05
0.458	36.75	43.56	38.22	42.56	36.84	41.42	37.80	42.30
0.496	35.71	42.79	37.41	41.95	36.02	40.66	36.54	41.81
0.534	35.29	42.17	36.64	41.24	35.97	40.02	35.82	41.15
0.572	34.86	41.42	35.71	40.46	34.13	39.22	35.45	40.42
0.611	34.27	40.53	34.94	39.71	33.40	38.44	34.68	39.81
0.649	34.05	39.53	34.09	38.88	32.55	37.50	34.32	39.00

**Table 4:** The payloads with different layers and images.

	**Peppers**	**Airplane**	**Baboon**	**Boat**	**Bridge**	**Couple**	**Crowd**	**Elain**
Layer 1	155,489	166,206	88,028	153,938	104,043	185,214	81,730	137,341
Layer 2	46,557	33,836	49,935	47,872	63,101	17,155	49,233	63,037
Layer 3			25,799		33,515		28,111	
Layer 4			13,002				15,029	
payload	202,046	200,042	176,764	201,810	200,659	202,369	174,103	200,378
	**Flower**	**Girl**	**Goldhill**	**Hat**	**Lake**	**Lena**	**Loco**	**Martha**
Layer 1	127,797	174,779	145,195	174,660	130,152	166,059	133,218	177,391
Layer 2	72,605	27,185	55,131	28,366	70,131	34,375	67,396	22,741
Layer 3					1,592			
Layer 4								
payload	200,402	201,964	200,326	203,026	201,875	200,434	200,614	200,132

**Table 5:** Parameters of each layer.

Images and Parameters		Parameters of Each Layer								
		1	2	3	4	5	6	7	8	9
Lena	*A*	1	−2	3	−2	3	−3	4	3	−4
	*B*	0	−2	8	−2	3	0	6	−4	10
	*k*	1	2	1	1	1	1	1	1	1
Airplane	*A*	1	−1	−1	1	-	-	-	-	-
	*B*	0	0	−3	6	-	-	-	-	-
	*k*	1	3	1	1	-	-	-	-	-
Boat	*A*	1	−2	−2	−2	2	−4	−3	−2	−2
	*B*	0	0	8	4	2	−6	−8	8	8
	*k*	1	1	1	1	1	1	1	1	1

**Table 6:** Experiment compared with Chang’s method.

Images		Chang’s Method	Proposed Method	Compare
Lena	Embedded Data	62,784	99,767	+36,983
	Extra Information	68,288	273	−68,015
	PSNR (dB)	43.73	49.85	+6.12
Airplane	Embedded Data	74,656	99,909	+25,253
	Extra Information	56,416	133	−56,283
	PSNR (dB)	43.71	49.14	+5.43
Boat	Embedded Data	48,888	69,763	+20,875
	Extra Information	82,184	277	−81,907
	PSNR (dB)	43.22	49.61	+6.39

**Table 7:** Performance comparison among different reversible information hiding methods.

Image		Hung’s Method	Hong’s Method	Luo’s Method	Cai’s Method	Proposed Method
**Lena**	Payload	59,751	46,839	71,674	7964	100,040
	PSNR (dB)	48.64	49.19	48.82	62.16	49.85
**Airplane** **(F-16)**	Payload	66,465	64,863	84,050	21,300	70,042
						100,042
	PSNR (dB)	48.75	49.39	48.94	58.27	49.70
						49.14
**Boat**	Payload	37,938	29,824	-	2923	70,040
	PSNR (dB)	48.41	49.02	-	66.26	49.62

## Data Availability

Open-source image databases (Image Processing Place, https://www.imageprocessingplace.com/root_files_V3/image_databases.htm, accessed on 2 February 2022).
